# Cisplatin exhibits superiority over MMC as a perfusion agent in a peritoneal mesothelioma patient specific organoid HIPEC platform

**DOI:** 10.1038/s41598-023-38545-4

**Published:** 2023-07-19

**Authors:** Steven D. Forsythe, Richard A. Erali, Nicholas Edenhoffer, William Meeker, Nadeem Wajih, Cecilia R. Schaaf, Preston Laney, Cristian D. Vanezuela, Wencheng Li, Edward A. Levine, Shay Soker, Konstantinos I. Votanopoulos

**Affiliations:** 1 Wake Forest Institute for Regenerative Medicine, Winston-Salem, USA; 2 Wake Forest Organoid Research Center, Winston-Salem, USA; 3 Division of Surgical Oncology, Department of Surgery, Wake Forest Baptist Health, Medical Center Boulevard, Wake Forest University, Winston-Salem, NC 27157 USA; 4 Department of Pathology, Wake Forest Baptist Health, Winston-Salem, USA

**Keywords:** Cancer models, High-throughput screening, Double-strand DNA breaks, Mesothelioma, Tumour heterogeneity

## Abstract

Peritoneal mesothelioma (PM) is a rare malignancy with poor prognosis, representing about 10–15% of all mesothelioma cases. Herein we apply PM patient-derived tumor organoids (PTOs) in elucidating personalized HIPEC responses to bypass rarity of disease in generating preclinical data. Specimens were obtained from PM patients undergoing cytoreductive surgery with HIPEC. PTOs were fabricated with tumor cells suspended in ECM-hydrogel and treated with HIPEC regimen parameters. Viability and characterization analyses were performed post-treatment. Treatment efficacy was defined as ≥ 50% viability reduction and p < 0.05 compared to controls. From October 2020 to November 2022, 17 tumors from 7 patients were biofabricated into organoids, with 16/17 (94.1%) sites undergoing comparative 37° and 42° treatments with cisplatin and mitomycin C (MMC). Hyperthermic cisplatin and MMC enhanced cytotoxicity which reduced treatment viability by 25% and 22%, respectively, compared to normothermia. Heated cisplatin displayed the greatest cytotoxicity, with efficacy in 12/16 (75%) tumors and an average viability of 38% (5–68%). Heated MMC demonstrated efficacy in 7/16 (43.8%) tumors with an average treatment viability of 51% (17–92.3%). PTOs fabricated from distinct anatomic sites exhibited site-specific variability in treatment responses. PM PTOs exhibit patient and anatomic location treatment responses suggestive of underlying disease clonality. In PM organoids cisplatin is superior to MMC in HIPEC.

## Introduction

Peritoneal mesothelioma (PM) is a rare malignancy and accounts for an estimated 10–15% of the 3000 mesothelioma cases in the US every year^1,2^. 5-year overall survival rates exceeding 60% have been reported in patients treated with complete macroscopic cytoreductive surgery (CRS) followed by hyperthermic intraperitoneal chemotherapy (HIPEC)^3–6^. Systemic chemotherapy and immunotherapy treatment decisions in PM are largely based on studies performed in its pleural counterpart^7,8^.

The low incidence of PM creates challenges in generating preclinical data regarding new treatments. Patient-derived tumor organoids (PTOs) are three-dimensional ex-vivo models that have been used to accurately reconstruct the cellular representation of individual patient tumors inclusive of intra- and inter-patient clonality, that is applicable to both cancers with high incidence as well as rare primaries. We have previously reported the feasibility of utilizing PTOs in a pre-clinical setting for several malignancies including appendiceal, colon, sarcoma, Merkel cell, melanoma, and mesothelioma^9–15^.

In this current study, we demonstrate the role of PTOs in studying patient specific variations in organoid responses to intra-peritoneal chemotherapy in PM patients undergoing CRS/HIPEC. We also characterized treatment response heterogeneity amongst anatomically distinct sites from individual patients. We hypothesize PM PTOs can be used to generate preclinical personalized data on responses to treatment without inhibition by the prohibitively low incidence which prospective trials are dependent on.

## Results

### Patient and tumor characteristics

Seventeen patient tumors were collected from seven patients during the study period from October 2020 to November 2022 (Table 1).

**Table 1:** Clinical correlation of PTO data with disease progression and overall survival (OS).

Patient	Tumor subtype	Resection status	HIPEC drug	Prior treatment(s)	Time to progression	Overall survival	Current clinical status
1	Epithelioid	CC1	MMC	None	N/A	22 months	AWD—stable
2	Epithelioid	CC2	None	Carboplatin/Bevacizumab/Pemetrexed	4 months	20 months	AWD—Adjuvant Immunotherapy
3	Epithelioid	CC1	Cisplatin	None	12 months	14 months	AWD—Adjuvant Immunotherapy
4	Epithelioid	CC2	Cisplatin	CRS/HIPEC × 2 (Cisplatin)	2 months	7 months	AWD—Adjuvant Immunotherapy
5	Epitheloid	CC0	None	Carboplatin/Pemetrexed CRS/HIPEC (MMC) HITEC	N/A	1 month	AWD—Adjuvant Immunotherapy
6	Biphasic	CC0	Cisplatin	None	N/A	5 months	DOD
7	Well Diff. Papillary w. invasive foci	CC1	Cisplatin	Carboplatin/Pemetrexed	15 months	15 months	AWD—stable

AWD, alive with disease, either stable or on adjuvant therapy; DOD, died of disease; HITEC, Hyperthermic intra-thoracic chemotherapy.

**Figure 1:**
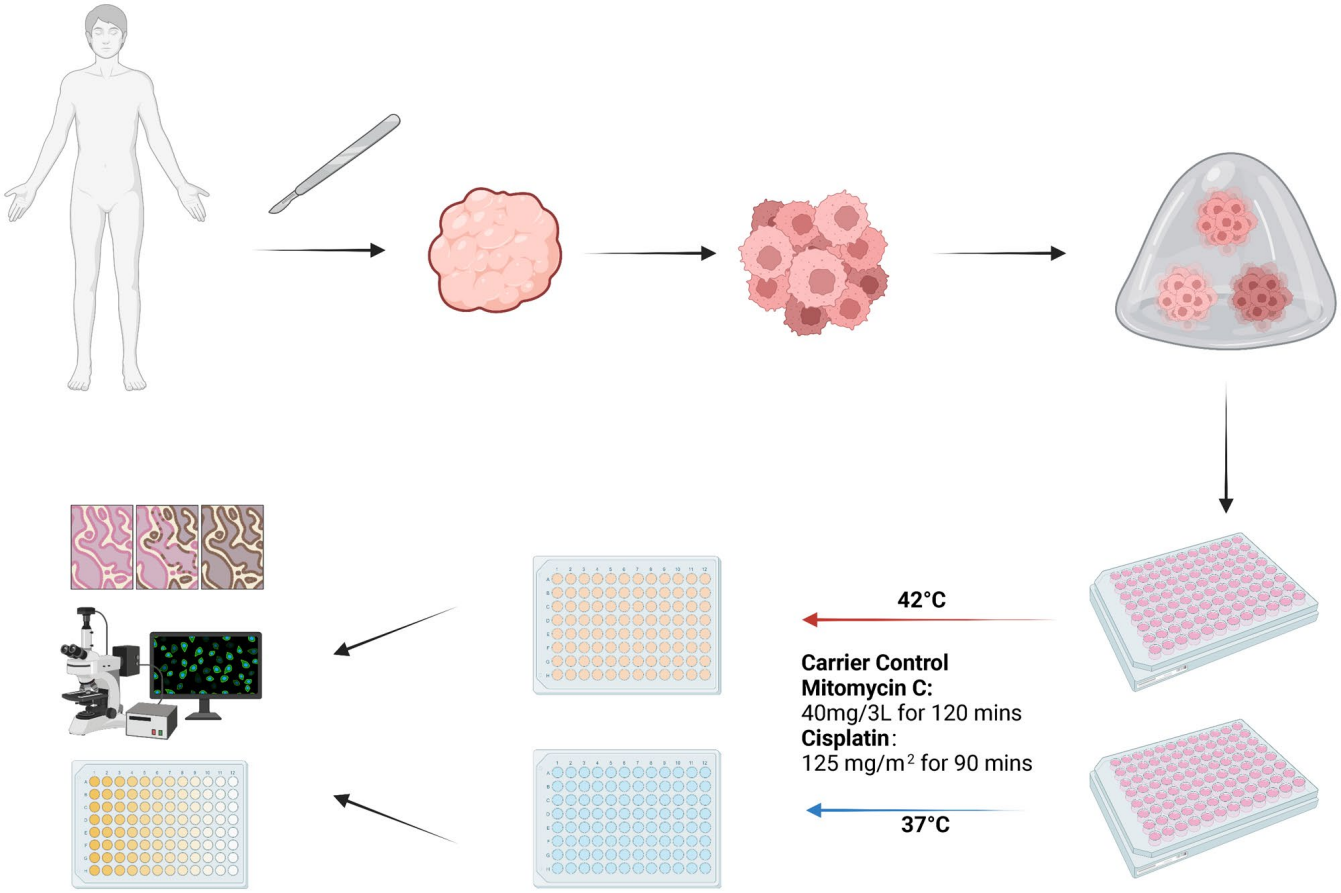
Fabrication of mesothelioma tumor organoids for HIPEC testing. Created with www.BioRender.com (created June 2023).

All tumors provided enough cells for HIPEC regimen comparison, although one PTO group was not sufficiently viable for testing. After biofabrication, PTOs were cultured for 7 days, treated with HIPEC regimens, and cultured for an additional 72 h prior to viability testing. Overall, results from each treatment were available within 10 days from tissue harvesting (Fig. 1).

### Cisplatin demonstrates superior HIPEC cytotoxicity when compared to mitomycin C

**Figure 2:**
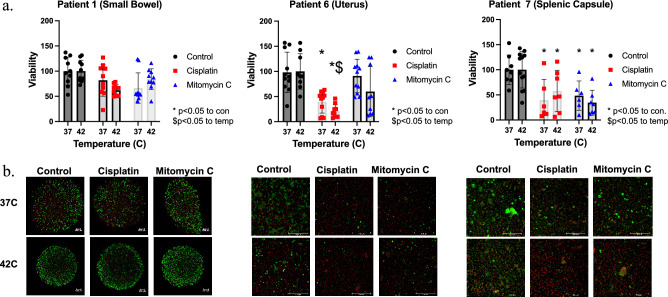
PTO sets demonstrate variable response to treatment in different patients using (**a**) Cell-Titer Glo and (**b**) LIVE/DEAD Imaging where green, living cells and red, dead cells.

**Figure 3:**
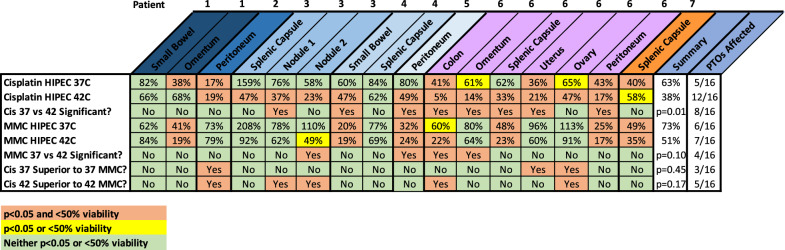
Summary of all PTO Cell-Titer Glo© results.

Patient derived organoid sets were treated with either cisplatin or mitomycin (MMC) based on clinically utilized regimens^5^. All viable PTO sets were treated under hyperthermic (42 °C) and normothermic (37 °C) conditions and response to treatment was recorded at the level of individual patients (PTO sets) as well as at the level of the entire cohort by pooling the overall response rate between patients (Figs. 2 and 3). Analysis demonstrated personalized variability between patients (Fig. 3 and Supplemental Fig. 1). Hyperthermia significantly increased the cytotoxicity of cisplatin (pooled analysis viability, 38.3% at 42 °C vs 62.6% at 37 °C, p = 0.02) and demonstrated a trend towards significance with MMC (50.6% at 42 °C and 73.3% at 37 °C, p = 0.10). However, despite the hyperthermia derived increase in cytotoxicity in both groups at the cohort level, the benefit observed with hyperthermic cisplatin was more notably pronounced at individual patient level, with 8/16 (50%) PTO sets gaining a significant boost in cytotoxicity when compared to normothermic temperature treatments, while the benefit for MMC was observed in only 4/16 (25%) PTO sets. When comparing the pooled responses to hyperthermic cisplatin and MMC, there was no statistically significant difference in cytotoxicity between the two agents (38.3% vs 50.6%, respectively, p = 0.10). However, when comparing individual patient organoid results, heated cisplatin was significantly more effective in 5/16 (31.3%) PTO sets, whereas heated MMC was significantly more effective in only 2/16 (12.5%) of PTO sets (Patient 1 Omentum and Patient 5 Peritoneum). LIVE/DEAD imaging emphasizes these differences for HIPEC sensitive and resistant PTO sets (Fig. 2b).

### Organoid HIPEC response demonstrates variability between tumors from the same patient

**Figure 4:**
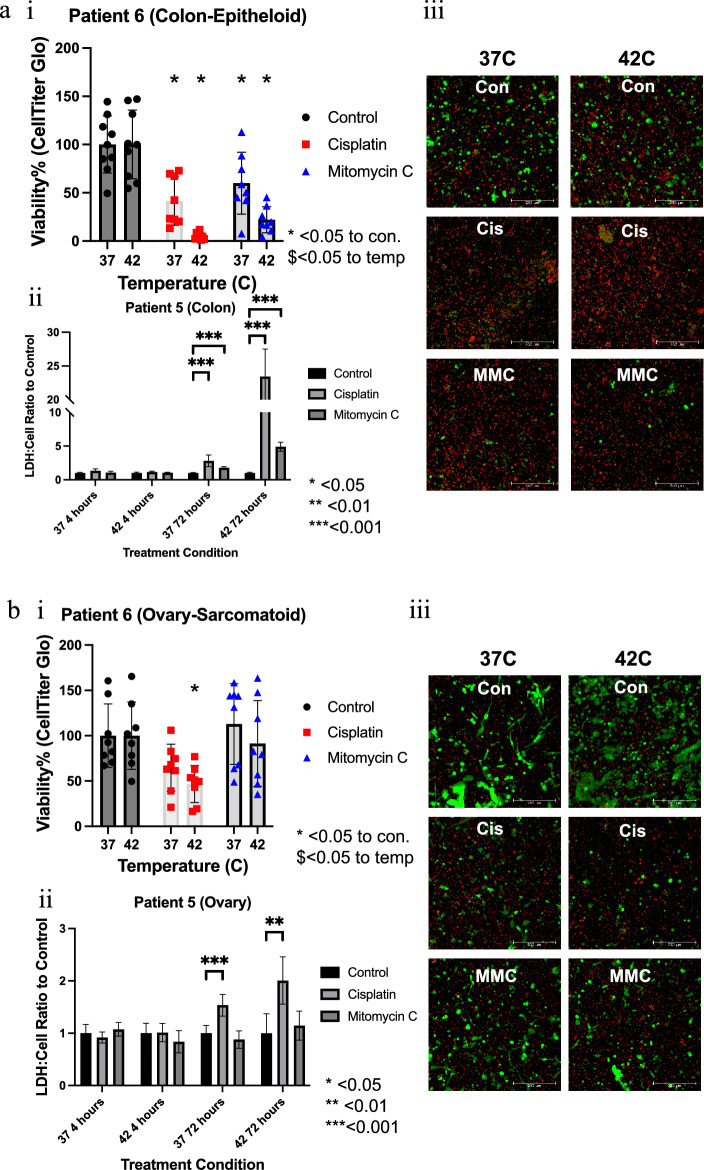
Variability in PTO treatment response between mesothelioma implants from Patient 6 with biphasic histologic subtype, suggesting heterogenous tumor cell populations when comparing implant tumor cells from (**a**) colon (epithelioid) and (**b**) ovary (sarcomatoid) using analyses (**i**) Cell-Titer Glo, (**ii**) LDH quantification and (**iii**) LIVE/DEAD imaging.

Tumor biology in peritoneal disease presents unique challenges because aggressive peritoneal tumor deposits are likely to harbor different subpopulations of cells than other sites of metastases. CRS/HIPEC may be able to provide the opportunity to anatomically map treatment resistant clones. To investigate this possibility, individual patient implants from anatomically distinct locations were compared for their response to HIPEC therapy (Fig. 4). The cytomorphology shows different features between the two locations, with PTOs from metastatic PM to the surface of the colon demonstrating an epithelioid morphology while PTOs from metastatic PM to the ovary show ovoid to sarcomatoid spindle morphology (Fig. 4 and Supplemental Fig. 2a). Additionally, the cytomorphologies of PTOs were identical to their matched tumor tissues (Supplemental Fig. 2).

Comparison of PM implants derived from the colon and ovary showcase the differences in HIPEC response, with the colon derived PTO set highly sensitive to both normothermic and hyperthermic cisplatin and MMC treatment conditions (Fig. 4ai), whereas the ovary PTO set was resistant towards MMC treatment and was only sensitive to heated cisplatin (Fig. 4bi). Additionally, cellular stress testing quantified by LDH analysis at 4 and 72 h demonstrated a large increase in measurable cellular stress markers for the colon implant PTOs at 72 h, indicating remaining cellular populations were in a high state of mitochondrial dysfunction (Fig. 4aii); notably this increase was also seen in the heated cisplatin treated ovarian implant PTO group but at a markedly decreased number when compared to colon implant PTOs.

### Increased DNA damage response demonstrates site specific treatment response differences

**Figure 5:**
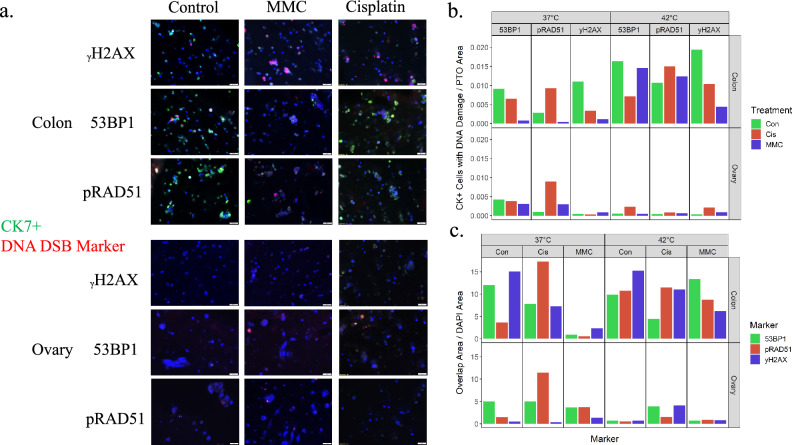
(**a**) Increased localization of DNA damage response to nucleus. Figure demonstrates the increase of DNA damage markers including 53BP1, phosphorylated RAD51 and yH2AX to the nucleus (DAPI), suggesting increased damage response in these cells. (**b**) Overlap of DNA damage markers (TxRed) in CK7+ expressing cells (FITC), displaying total number of CK7+ cells with DNA damage. (**c**) Overlap of DNA damage on nucleus (DAPI), displaying total number of cells with measurable DNA damage.

The addition of heat to chemotherapy treatment as performed in HIPEC leads to many altered cell responses, including DNA DSB repair^16^. To analyze this mechanism in PTOs, whole organoid analysis of DNA damage markers including γH2AX DNA binding, activation of p53-binding protein 1 (53BP1) and phosphorylation of DNA repair protein RAD51 homolog 1** (**RAD51) complexes, all members of the DNA double stranded break pathway, was performed in organoids at four hours post-treatment (Fig. 5). Cytokeratin 7 (CK7) was chosen to select for tumor cells as it is highly expressed in mesothelioma cancer cells across patients^17^. Analysis of total DAPI nuclear signal demonstrated similar cell quantification between the two organoid populations, while a marked decrease in CK7+ cells was detected in the ovarian implant derived PTO populations, suggesting another subpopulation of tumor cells (Fig. 5a and Supplemental Fig. 3). In hyperthermic treated conditions for colonic implant PTOs, there was a large increase in the quantity of the DNA repair markers localized to the nucleus, suggesting an increase in activity related to DNA damage and early repair (Fig. 5b). In contrast, there was minimal increase in these markers in treated ovarian implant PTOs, suggesting little DNA repair response in these organoids, and thus lower potential efficacy of HIPEC regimens in these PTOs. Additionally, there was a marked increase in overlap of DNA damage makers within CK7+ cells in the hyperthermic treated PTOs derived from the colonic implants, suggesting these cells are more sensitive to this treatment group (Fig. 5c).

### PTO response correlation with available clinical data

Establishing clinical correlation with peritoneal disease remains a challenge due to variable levels of completion of CRS, impact of residual disease on progression, limitations of imaging modalities and site-specific differential response to both HIPEC and systemic treatment. Nevertheless, available data are reported for completion purposes.

Patients 1 and 3 had both CC1 CRS (Table 1). Patient 1 was perfused with MMC and remains without disease 22 months later (his post perfusion omentum organoids with MMC showed viability of 19%), while patient 3 was perfused with cisplatin with best PTO post perfusion viability of 23% and remained without progression for a year (Fig. 3). Patient 4 had a CC2 incomplete CRS, was perfused with cisplatin with best post perfusion organoid viability of 47% and was dead at 7 months post-surgery. Patient 6 (biphasic) provided organoids that exhibited a range of 5–33% post perfusion viability for the predominant epithelioid colon lesions and a 47% viability for the sarcomatoid ovarian lesion. The patient despite optimal CRS (CC0) was dead at 5 months post-surgery. Patient 7 (well differentiated papillary) was perfused, as the final pathology was unknown at the time of surgery. Organoids revealed a post perfusion cellular viability to cisplatin of 58% that is expected given the biologic behavior of the specific mesothelioma subtype.

## Discussion

Over the last 25 years, increased understanding of tumor biology along with improved patient selection and optimized perfusion protocols have established CRS/HIPEC as the gold standard in the treatment of PM with 5-year survival rates reaching 65% in patients who have achieved complete macroscopic cytoreduction^4–6,18–21^. However, the rarity of disease remains a challenge in generating sufficiently powered prospective randomized clinical trials while retrospective studies rely on inclusion of patients treated over decades to sufficiently power statistical models^6,22,23^. Our work is predicated on harnessing PTOs to overcome the cohort limitations inherent to rare cancers, including PM. Building on our previous success in modeling rare cancer primaries using PTOs^9–13,24^, we herein describe the successful application of patient derived organoids to model HIPEC treatment in peritoneal mesothelioma attempting to quantify differential responses to treatment between patients as well as the impact of clonality in the differential response of anatomically distinct lesions derived from the same individual.

Hyperthermia improved the cytotoxicity of both cisplatin and MMC by further reducing post-treatment viabilities 25% and 22%, respectively, compared to normothermia. Mechanisms behind perfusates used in HIPEC generally result in the creation of DNA adducts, causing DNA double stranded breaks (DSB), and ultimately cell death^25^. The addition of hyperthermia has been theorized to cause a range of effects on treatment efficacy, including increased therapy uptake into cells, increased integration into DNA strands, and altered DNA repair^26,27^. In our PTO model, the increase in DNA damage can be tumor site specific and may be increased in organoids undergoing heated treatment. Following treatment, increases in yH2AX formation and the two DNA DSB repair pathways, including homologous recombination protein pRAD51 and non-homologous end joining protein 53BP1 complexes, suggests widespread DNA damage response in these groups, leading to cell death and reduction in the PM cells as seen in our viability results. Therefore, mapping DNA damage responses may serve as an early indication of treatment effectiveness and a functional measurement of HIPEC efficacy while demonstrating the value of PTOs as a tool for further analysis of these pathways.

Overall, PM PTOs demonstrated personalized variability in treatment responses, with hyperthermic cisplatin resulting in the lowest average post-treatment viability of all four treatment arms, with significantly greater benefit over normothermic cisplatin. We observed greater efficacy in heated cisplatin treatments compared to heated MMC (38% vs. 51% average post-treatment viabilities, respectively), which aligns with historic retrospective survival analysis of PM patients treated with cisplatin-based CRS/HIPEC^4,5,28^. Blackham et al., found patients perfused with cisplatin experienced median survival of 40.8 months compared to 10.8 months for those perfused with MMC^5^ and additional evidence for cisplatin has since been established^28^. The administration of cisplatin based neoadjuvant chemotherapy in Patients 2 and 7 did not appear to promote treatment resistance clones, since most treatment conditions still resulted in significant reduction in post-treatment viabilities. The short timeframe between completion of neoadjuvant treatment and CRS/HIPEC may not be long enough to allow for underrepresented resistant clones to propagate and manifest themselves as dominant clones in the patient’s response. While patients with well differentiated papillary subtype are considered for CRS only upon rapid progression, perfusate selection might be important for those with invasive features. Heated cisplatin appeared to offer greater overall cytotoxicity over MMC, but interestingly in the well differentiated papillary subtype with invasive features, heated MMC provided the greatest benefit. While current data have previously demonstrated a survival benefit of cisplatin over MMC, some patients within the cohort will still do better with MMC, therefore PTOs can potentially help optimize HIPEC perfusates on an individual patient basis. Thus, PM PTO models may be beneficial in understanding the impact of peri-operative systemic therapy, potential symptomatic relief of ascites and subtype-based perfusate selection.

Clonal development creates unique cellular populations whose genetic and phenotypic behavior vary greatly^29,30^. Notably, our PTO model preserved the phenotypic and histologic architecture of Patient 6’s biphasic subtype between the colon and ovarian metastatic sites, which allowed us to observe the heterogeneity in treatment responses between sarcomatoid and epithelioid components. LDH production and DSB pathway activation indicated the sarcomatoid ovarian tumor metastasis was not well responding to HIPEC treatment, suggesting a treatment resistant clone which may lead to tumor recurrence. Similarly Patient 4 had co-existing implants that were both responsive and not responsive to perfusion. Understanding the effect of clonality on patient outcomes, may not only help predict patient response to therapy, but also aid in understanding mechanisms of treatment resistance and allow for the selection of second line treatments. Traditional ex-vivo models of cancer including two-dimensional cell culture and patient derived xenograft models fail to properly maintain cellular signaling, are subject to inaccurate results due to the presence of xenobiologic factors, and often suffer from low establishment rates^31^. Organoids manage to maintain the advantages of both systems while avoiding the weaknesses of either, allowing for ideal experimental control and biological relevance for clinical applications. Furthermore, PTO identification of treatment resistant clones may help improve perfusate selection in patients undergoing repeat CRS/HIPECs, especially considering repeat CRS/HIPECs can significantly prolong survival in carefully selected patients^32^.

Correlating clinical data with organoid response provides with an opportunity to improve our understanding on disease biology at the level of the individual patient. The multiclonal nature of peritoneal disease along with variability in the level of CRS completion represent unique challenges in providing a definition of what constitutes a responding and non-responding tumor. Despite the above we observed that patients with organoid responses, where approximately 20% of the cells remained alive after perfusion were exhibiting lengthier time to progression while in cases where close to 50% of the cells were still viable post perfusion the outcome was guarded. As more data are accumulated over time, an individualized prognostic model may potentially be developed not only for mesothelioma but for all peritoneal primaries at the time of diagnosis and prior to HIPEC administration.

While the results of this study support CRS/HIPEC in the treatment of PM, there are some limitations. The rarity of PM and further division into subtypes resulted in a modest study cohort. PTOs may be beneficial in overcoming this limitation as preclinical companion diagnostic platforms, while ex-vivo generated drug data may supplement our current clinical knowledge. Clinical correlation in the setting of peritoneal disease remains difficult due to the heterogeneity of disease between often innumerable lesions. Even though sequencing data were not obtained, we hope the anatomic variations as well as pooled responses presented within this study will serve as a foundation for future study design and discussion in bringing PTOs to the forefront of translational research in peritoneal disease.

In conclusion, cisplatin demonstrates superiority and increased efficacy in hyperthermic conditions in PM when compared to mitomycin C. Clonality variations between spatially distinct lesions should be factored in the design of future clinical research models. Patient-derived tumor organoids can serve as a platform to test clinical therapeutic sensitivity in peritoneal mesothelioma and may help elucidate both inter- and intra-patient heterogeneity.

## Methods

All methods were performed under institutional guidelines in accordance with Wake Forest Baptist Health policies.

### Tumor biospecimen and cell processing

Specimens were procured under an IRB protocol approved by the Wake Forest Human Research Institutional Review Board. Informed consent was obtained from all the PM patients undergoing CRS/HIPEC procedures. The specimens were placed in RPMI and transferred to the laboratory within a 2-h framework.

Tumor dissociation and organoid formation were performed within 24 h. Tumors were rinsed twice for five minutes each in a wash solution of DPBS with 100 U/mL penicillin/streptomycin, 5 µg/mL gentamicin (G1272, Sigma Aldrich, St. Louis, MS) and 5 µg/mL amphotericin B (A2942, Sigma). Tumors were minced finely, removing fat, necrotic, and mucinous tissue. Tissue was placed into a 15 mL conical containing DMEM low glucose with no serum, 100,000 CDA units/mL Collagenase HA 200 Wünch Unit (001-1050, VitaCyte, Indianapolis, IN), 22,000 NPA units/mL BP Protease (003-1000, VitaCyte), and 100 mM *N*-acetyl l-cysteine (A9165, Sigma), with the total volume equal to 3 mL solution per gram of tissue. The conical was agitated at 37 °C until the tumor was dissociated, with two hours as the maximum time allowed. After the tumor was dissociated, an equal volume of cold RMPI-10 was added to quench remaining enzymatic activity, and the tumor solution was filtered through a sterile vacuum filtration kit with 60 µM pore size (SCNY0060, Millipore) to remove remaining undigested pieces of tissue and centrifuged. BD Pharm Lyse (555899, BD Biosciences) was then performed on the cell pellet according to company protocol. Remaining cells were counted and ready for use in organoid fabrication.

### Organoid formation

Organoids were formed by suspending cells in a thiolated hyaluronic acid (HA) (Glycosil, ESI-BIO, Alameda, CA) and methacrylated collagen (PhotoCol, Advanced BioMatrix, Carlsbad, CA) hydrogel. Thiolated HA was dissolved at 1% w/v each in deionized water containing 0.1% w/v Irgacure D-2959 (410896, Sigma) and combined with 3 mg/mL methacrylated collagen in a 1:3 ratio. For organoid formation, the hydrogel solution was used to resuspend cells at 10 × 10^6^ cells/mL. The hydrogel solution/cell suspensions were placed into the wells of a 96 well non-tissue culture treated plate at 5 µL per well. Each organoid was then exposed to UV light from a BlueWave 75 V.2 UV spot lamp (Dymax Corp., Torrington, CT) for one second per construct. The constructs were then covered with 200 µL media, with composition of Advanced DMEM-F12 (12634010, ThermoFischer Scientific), 5% Heat Inactivated Fetal Bovine Serum (10082147, ThermoFischer), 50 ng/mL EGF (PHG0313, ThermoFisher Scientific), 2 mM Glutamax, 1% penicillin/streptomycin, 10 μM Y-27632 (S1049), 10 μM SB202190 (S1077, SelleckChem). Organoids were maintained in an incubator (37 °C, 5% CO_2_) for seven days to ensure successful cell culture until treatment began.

### HIPEC treatment

After seven days, media was removed from organoid culture and replaced in parallel with 37 °C and 42 °C treatments consisting of untreated control media and 40 mg/3 L mitomycin C (M4287, Sigma Aldrich) for 120 min in temperature matched incubators. Similarly, another treatment of 125 mg/m^2^ cisplatin (232120, Sigma-Aldrich) was performed for 90 min in matched conditions. PTOs were treated with drug concentrations achieved in the peritoneal cavity of an average patient, with a body surface area of 1.9 M^2^, perfused with a volume of 3 L of perfusate. Dose and perfusion times were determined through previous institutional cohort studies^5^. After the elapsed time, the treatment was removed from each well and each well was rinsed with fresh media added to replace the wash solution. Organoids were incubated for 72 h post-treatment before viability assays were performed.

### Cell TiterGlo viability assay

After 72 h 3D Cell-Titer Glo Luminescent Cell Viability Assay (G968B, Promega, Madison, WI), was performed according to manufacturer’s instructions. The entire contents of each well were added to Costar White Polystrene 96 well Assay Plate (3912, Corning, NY) wells and read on a Veritas Microplate Luminometer (Turner BioSystems, Sunnyvale, CA). Values were averaged for experimental groups and analyzed using Graph Pad Prism© v.8 (Graphpad, La Jolla, CA).

### LDH-GLO viability assay

LDH-GLO (J2381; Promega) cytotoxicity assay was performed by preserving culture medium from post-treatment timepoints of 4 h and 72 h, then running the assay on the same day according to manufacturer specifications to measure lactose dehydrogenase (LDH) enzymatic activity. Well contents for both assays were transferred to a Costar White Polystyrene 96 well Assay Plate and analyzed with a Veritas Microplate Luminometer.

### LIVE/DEAD analysis

Media was removed from wells containing treated organoids and viability was assessed by LIVE/DEAD Viability/Cytotoxicity Kit assays (L3224, Invitrogen, Carlsbad, CA), with organoids incubated for two hours. Imaging was performed by macro-confocal microscopy (Leica TCS LSI, Leica, Wetzlar, Germany) and composite images were created with ethidium homodimer-1 red fluorescence indicating dead cell nuclei and calcein AM green fluorescence indicating viable cells.

### Immunohistochemistry and image analysis using Visiopharm

Following completion of in vitro HIPEC, treated organoids were fixed for 4 h in 4% paraformaldehyde. Organoids were processed, paraffin embedded, and sectioned for staining. Unstained slides underwent antigen retrieval in a pH 6 citrate buffer solution prior to blocking with Dako Protein Block for 30 min. Fluorescent IHC staining was performed with antibodies for CK7 (ab9021, Abcam, Cambridge, UK), RAD51 (ab133534, Abcam), 53BP1 (ab175933, Abcam), and γH2AX (ab11174, Abcam) with overnight staining at 4 °C. After incubation overnight at 4 °C, appropriate species reactive secondary antibodies at 488 (FITC) or 594 (TxRed) (Biotium, Fremont, CA) were applied to samples for 1 h at a 1:1000 dilution. Sections were then incubated with DAPI for 5 min prior to finalization with coverslipping. An Olympus BX-63 upright fluorescent microscope was used to image the sections.

Images were imported into Visiopharm imaging processing software and regions of interest (ROIs) were drawn around each organoid, excluding unwanted histological artifacts such as folded regions and air bubbles. Images were normalized to background by ROI to reduce lighting and staining variance between regions. Visiopharm was then used to digitally label DAPI, FITC, and TxRed using unique thresholds for each channel and for each image as set by a human rater. Measurements of total ROI area, DAPI area, FITC area, and TxRed area were taken and exported for analysis. FITC-TxRed overlap was calculated by labeling both FITC and TxRed with FITC prioritization and subtracting the remaining TxRed area resulting from this analysis from the total TxRed area measured previously.

### Statistical analysis

The data are generally presented as the means of the number of replicates ± the standard deviation. All data are graphed and analyzed for significance using a Student’s T-test. Pooled analysis of organoids utilized all individual organoid viability values in matching treatments to compare regimen efficacy. The authors defined successful treatments as those, which reduced tumor viability greater than 50% and reached significance of p < 0.05 compared to untreated control conditions. Data samples were eliminated from the experimental groups if they fell outside two standard deviations from experimental group averages. Sample sizes (n ≥ 5) were determined based on preliminary experiments. These sample sizes, with typically observed standard deviations, allowed statistical significance at p < 0.05 with statistical power greater than 80%.

## Supplementary Information

Supplementary Figures.

## Data Availability

Data for CellTiter-Glo 3D and LDH cytotoxicity assay results is available in the supplementary data. Further data is available upon request by emailing the corresponding author, Dr. Konstantinos Votanopoulos.
