# Supplementary material for: Cisplatin exhibits superiority over MMC as a perfusion agent in a peritoneal mesothelioma patient specific organoid HIPEC platform
Source: Sci Rep. 2023 Jul 19;13:11640. doi: 10.1038/s41598-023-38545-4 (PMC10356916; doi:10.1038/s41598-023-38545-4)
Supplement: Supplementary file 1 — Supplementary Figures. [file 41598_2023_38545_MOESM1_ESM.pdf]

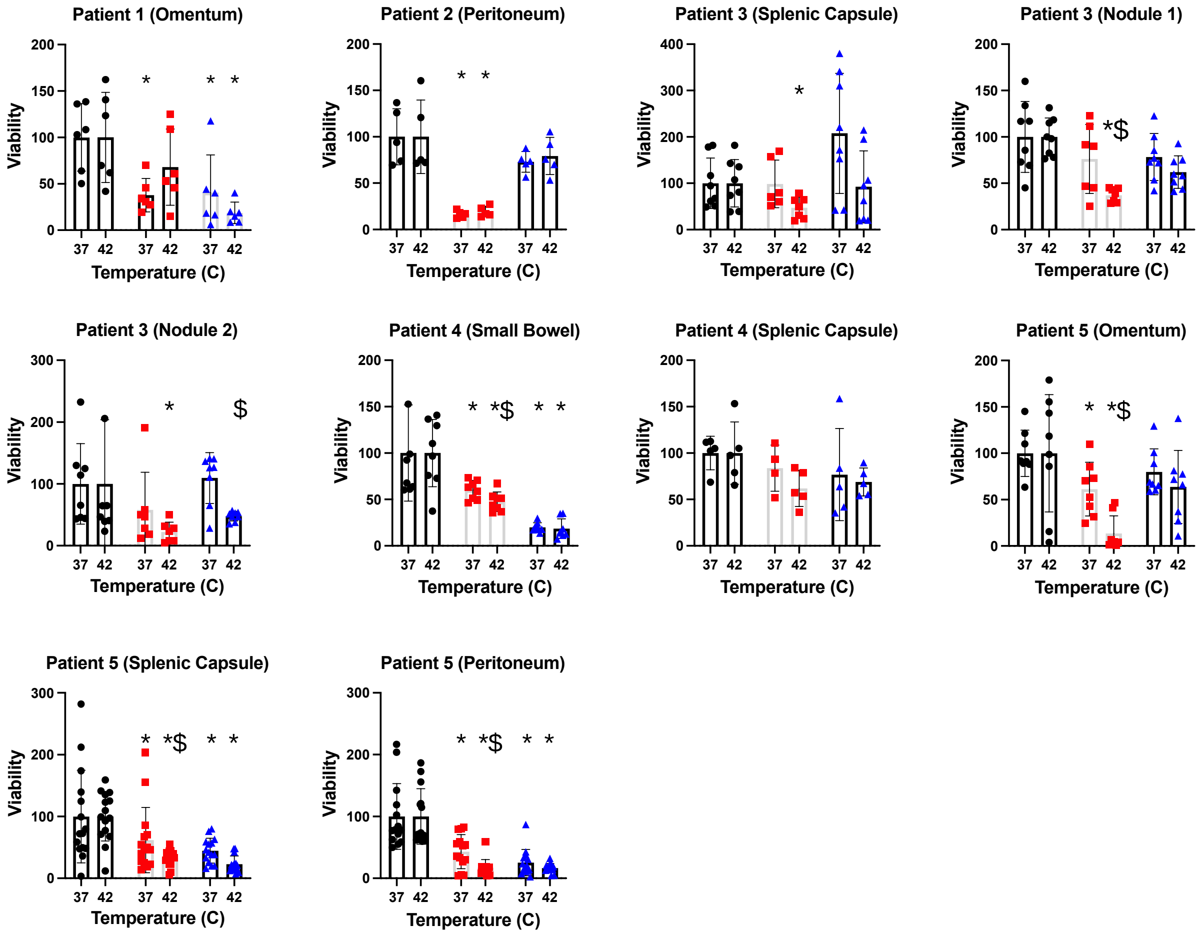

Supplementary Figure 1: Mesothelioma PTO responses to HIPEC. *=p<0.05 vs temperature matched control. $p<0.05 vs temperature matched counter condition.

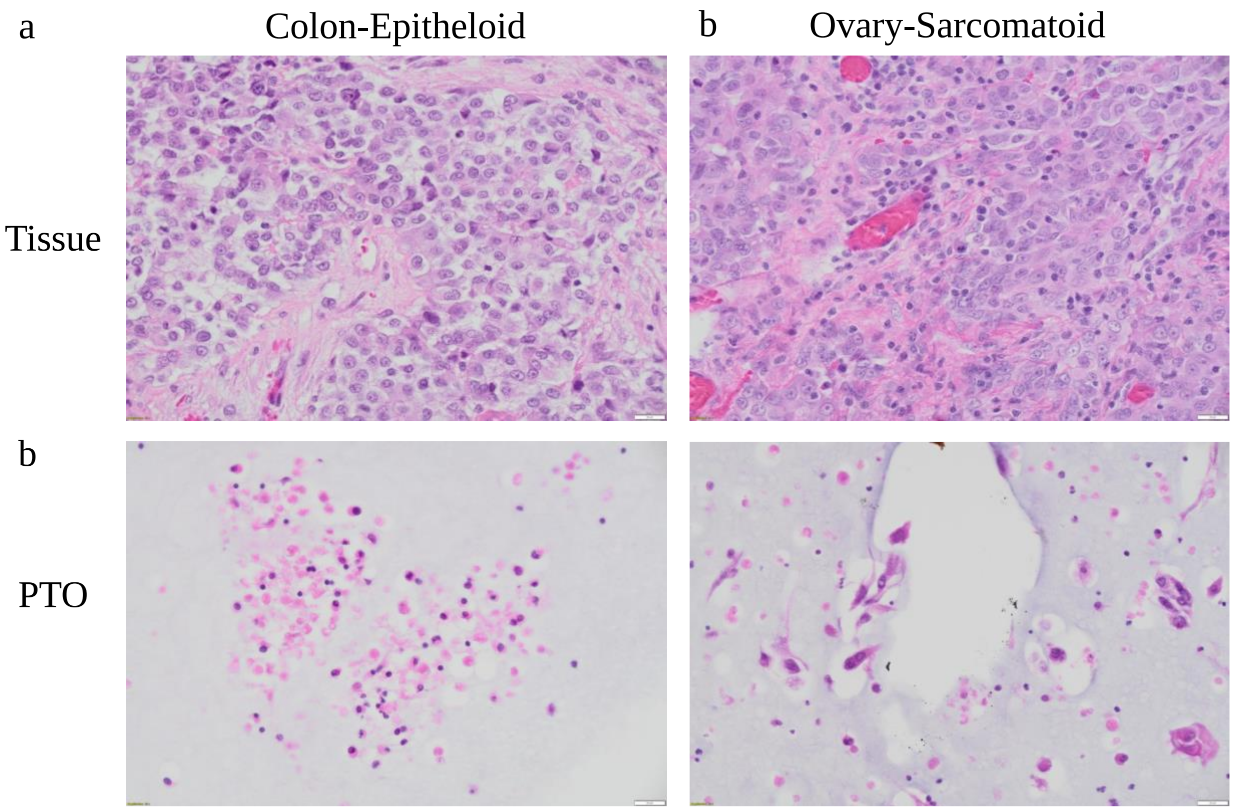

Supplementary Figure 2: Comparison of cells from a. colon (epitheloid) and b. ovary (sarcomatoid) tissues demonstrates phenotypic variation of the tumor cell populations with PTOs demonstrating similar cellular morphology as their matched donor tissue.

a

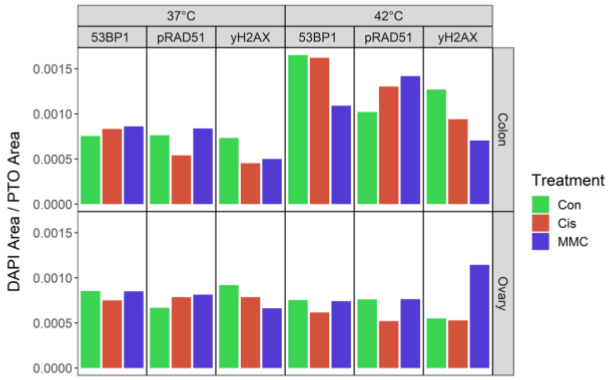

b

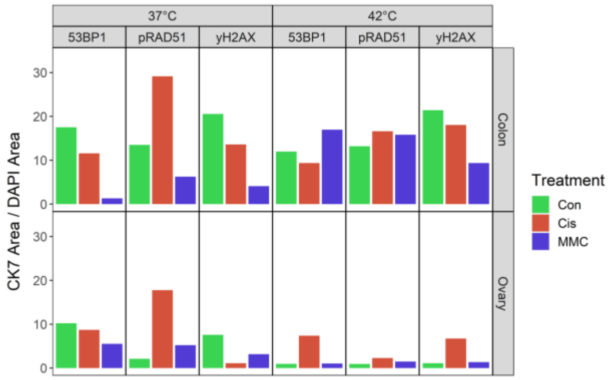

Supplementary Figure 3: a) quantification of cells comparing cell nuclei (DAPI) over PTO area and b) quantification of CK7+ cells (FITC) over PTO area.
